# Micromachined Resonant Frequency Tuning Unit for Torsional Resonator

**DOI:** 10.3390/mi8120342

**Published:** 2017-11-25

**Authors:** Jae-Ik Lee, Bongwon Jeong, Sunwoo Park, Youngkee Eun, Jongbaeg Kim

**Affiliations:** School of Mechanical Engineering, Yonsei University, 50 Yonsei-ro, Seoul 03722, Korea; sbrm@yonsei.ac.kr (J.-I.L.); jeongbw@gmail.com (B.J.); acidor@gmail.com (S.P.); yeun@yonsei.ac.kr (Y.E.)

**Keywords:** resonant frequency tuning, shaft-widening, shaft-holding, torsional resonator

## Abstract

Achieving the desired resonant frequency of resonators has been an important issue, since it determines their performance. This paper presents the design and analysis of two concepts for the resonant frequency tuning of resonators. The proposed methods are based on the stiffness alteration of the springs by geometrical modification (shaft-widening) or by mechanical restriction (shaft-holding) using micromachined frequency tuning units. Our designs have advantages in (1) reversible and repetitive tuning; (2) decoupled control over the amplitude of the resonator and the tuning ratio; and (3) a wide range of applications including torsional resonators. The ability to tune the frequency by both methods is predicted by finite element analysis (FEA) and experimentally verified on a torsional resonator driven by an electrostatic actuator. The tuning units and resonators are fabricated on a double silicon-on-insulator (DSOI) wafer to electrically insulate the resonator from the tuning units. The shaft-widening type and shaft-holding type exhibit a maximum tuning ratio of 5.29% and 10.7%, respectively.

## 1. Introduction

Micromachined resonators have a broad range of applications owing to their various advantages such as fast response, high sensitivity, small size, low power consumption, and low fabrication cost [1,2,3]. To ensure high and uniform performance, the micromachined resonators are supposed to have desired resonant frequencies. However, the resonant frequencies of the micromachined resonators often deviate from the intended value. One major source of this deviation is a dimensional error inevitably existing in fabrication process. It has been reported that relative tolerance in microfabrication could be as high as ±20% of the minimum feature size [4]. In addition, the cross section of fabricated microstructures might be trapezoidal instead of intended square shape due to an imperfect ion-reactive etching process [5]. The Microfabrication Laboratory at UC-Berkeley statically analyzed the resonant frequencies of 31 micromachined resonators, and reported a maximum discrepancy up to 4.9% [6]. It is also reported that the micromachined resonators generally have a deviation in resonant frequency between ±1%~±5% [7]. Operation environments such as pressure and temperature can also shift the resonant frequency of the resonators [8,9]. While the fabrication errors are constant after manufacture, environmental factors vary over time, which may cause frequent or continuous changes in resonant frequency.

Previous works for resonant frequency tuning can be categorized into two groups. The first approach is based on the permanent structural modification of the resonators, which is usually referred to as passive frequency tuning [6,10,11,12]. Passive frequency tuning can be accomplished by increasing the vibrating mass by the post-fabrication process such as pulsed laser deposition [6] and platinum deposition using a focused ion beam (FIB) [10]. Stiffness adjustment has also been presented by using polysilicon deposition [11] and FIB-machining [12]. The main advantage of the passive frequency methods is that they do not consume power to maintain the tuned state. On the other hand, the second approach does not rely on permanent structural modification, and therefore offers reversible and active tuning capabilities [13,14,15,16,17,18,19,20,21,22,23]. Previous works for active frequency tuning utilized the electrostatic spring effect [14,15], the geometry of the capacitors [16,17,18,19,20,21], the thermal stressing effect [22], and thermal expansion [23]. Only a few works have been reported regarding the frequency tuning of torsional resonators [14,24,25], such as designing angle limiters near the torsional spring [24] or inducing stress on bending flexures [25].

In this paper, we present two concepts for the resonant frequency tuning of torsionally-driven resonators [26]. The proposed methods are based on the micromachined frequency tuning unit, which is integrated together with electrostatic torsional resonator on the same chip. The separation of actuators for the tuning unit and resonator enables independent control over the amplitude and resonant frequency of the resonator, allowing continuous and repetitive tuning.

## 2. Design and Principle

**Figure 1:**
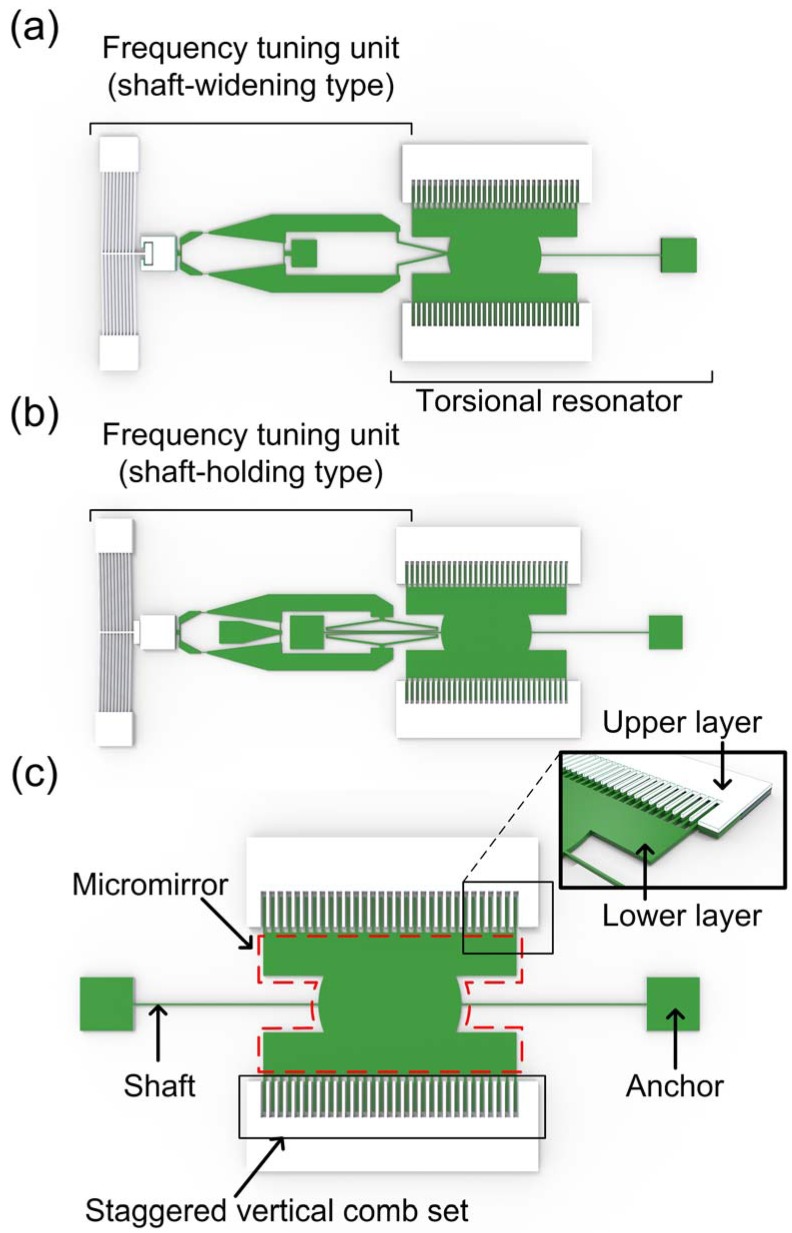
Schematics of frequency tuning units integrated with torsional resonators: (**a**) Shaft-widening type and (**b**) shaft-holding type. (**c**) Schematic layout of staggered vertical comb (SVC)-based torsional resonator. The micromirror is designed to optically read the torsional angle of the resonator. (Inset: close-up view of the SVC set defined on different silicon layers. Upper and lower layers are colored in white and green, respectively). Reproduced with permission from [26].

Our concept for resonant frequency tuning is based on the stiffness alteration of the torsional shaft either by widening the angle of tilted shafts, or by constraining the torsional motion of the shaft. Hereafter, the former and the latter are referred to as shaft-widening and shaft-holding, respectively. The schematics of the frequency tuning units integrated with torsional resonators are shown in Figure 1: (a) shaft-widening type, and (b) shaft-holding type. A staggered vertical comb (SVC) is utilized to actuate the torsional resonator, and a micromirror is designed to optically read the torsional angle of the resonator (Figure 1c). The resonator is torsionally-driven with respect to the torsional shafts by electrostatic force between two sets of fixed and movable comb fingers at different vertical positions, as shown in the inset of Figure 1c. The torsional resonator actuated by SVC sets is fabricated on two different levels of single crystalline silicon layers of double silicon-on-insulator (DSOI) wafer with fixed combs on upper layers (colored in white) and moving combs and mirrors on the lower layer (colored in green). The usage of silicon double layers allows self-alignment between the fixed and moving combs with simple fabrication [27]. 

**Figure 2:**
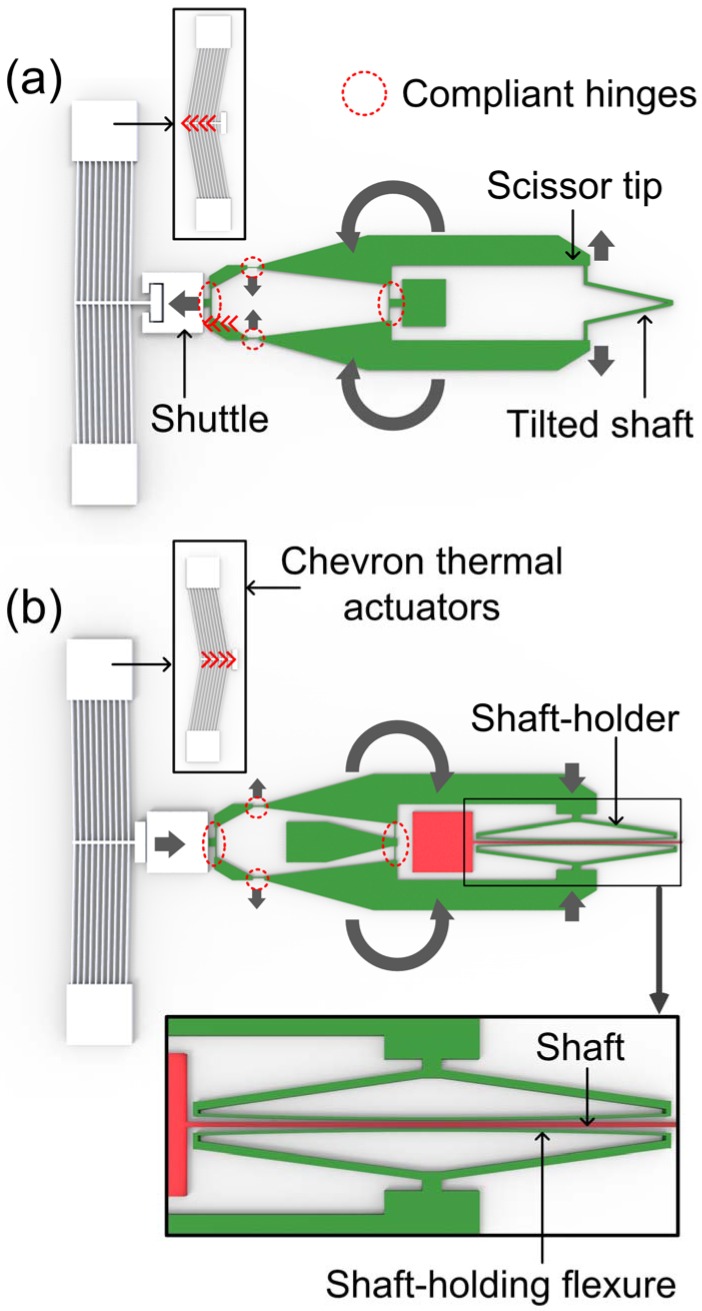
Design of (**a**) shaft-widening and (**b**) shaft-holding type frequency tuning units. Linear motion from the chevron thermal actuator (inset figure) is transformed and amplified to widen the gap between the tilted shafts (shaft-widening type), or to mechanically restrict the rotational motion of the shaft (shaft-holding type).

The frequency tuning units for shaft-widening are composed of a chevron thermal actuator [28] and a scissor mechanism part. The right ends of the scissor mechanism part (scissor tips) are connected to the tilted shafts of the torsional resonator (Figure 2a). The force from the chevron thermal actuator is used to drive the scissor mechanism. The scissor mechanism with three pairs of compliant segments is designed to amplify the stroke of the thermal actuator and to convert a linear displacement to a scissor blade-like symmetrical angular motion. As depicted in Figure 2a, when the shuttle is pulled leftward by the chevron thermal actuator, the scissor mechanism part is opened at the right end, widening the angle between the tilted shafts. This structural change increases the effective stiffness of the tilted shafts, resulting in an increase of resonant frequency.

**Figure 3:**
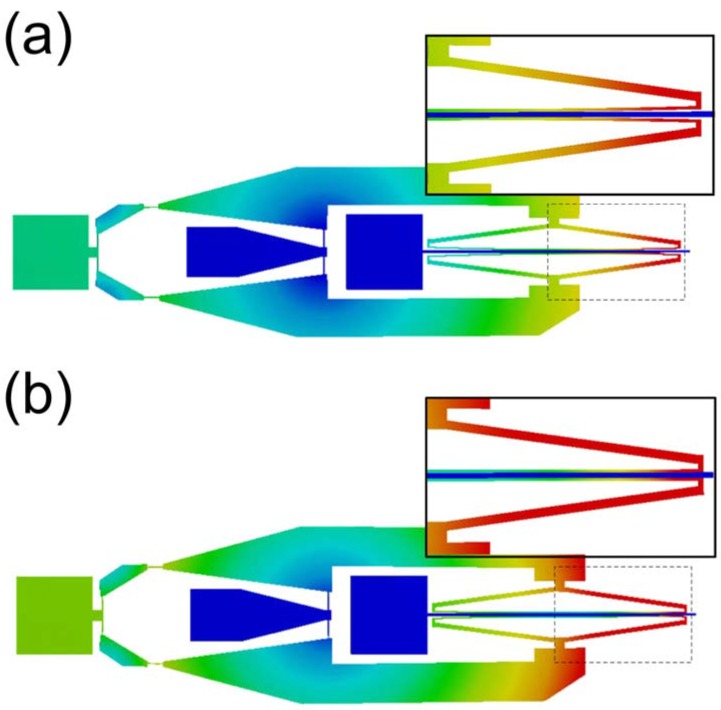
Schematic view of shaft-holding mechanism. (**a**) By the driving force from the chevron thermal actuator, the contact between the shaft-holding flexures and the torsional shaft starts to be formed from the middle of the torsional shaft; (**b**) As the shaft-holder moves further, the shaft-holding flexure is elastically deformed, resulting in a gradual increase in contact area.

The shaft-holding type also employed a chevron thermal actuator and the scissor mechanism, but the shaft-holders are attached to the scissor tips (Figure 2b). The chevron thermal actuator of the shaft-holding type is designed to push the shuttle rightward, as depicted in Figure 2b. Then, the scissor tips are closed at the right end, moving the shaft holders towards the torsional shaft of the resonator. The shaft holders are structured as a bow-like shape, by which the level of restriction upon the torsional spring would be continuously augmented as the shaft-holding flexure exhibits elastic deformation. When the mechanical restriction is initiated, the contact occurs at the middle of the torsional shaft resulting in a relatively lower tuning ratio (Figure 3a). As the shaft-holder moves further, full contact can be formed between the torsional spring and the holding flexure, extending the contact area to the segment close to the mirror (Figure 3b). In this way, both a high tuning ratio and a wide tuning range would be possible. 

**Figure 4:**
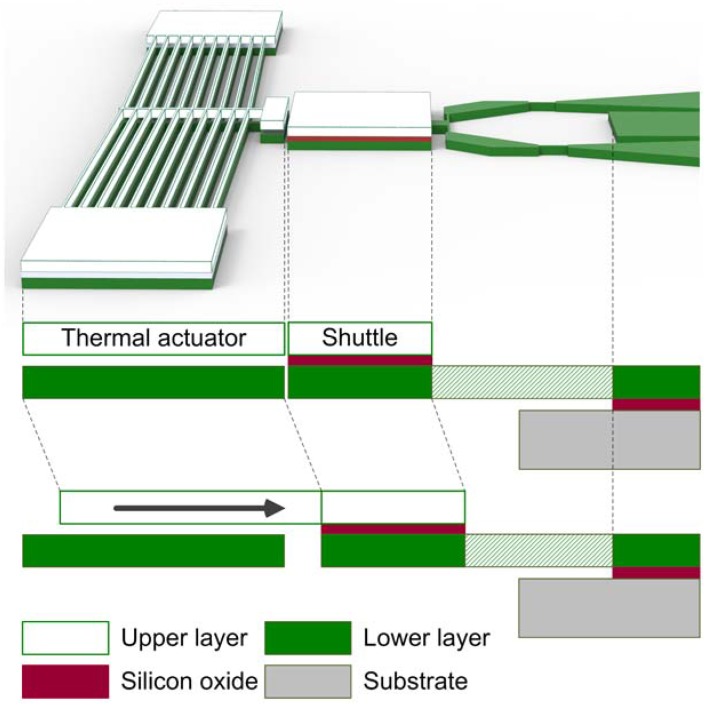
Electrical isolation design to avoid short circuit formation and charge leakage between the chevron thermal actuator and the electrostatic actuator.

One of critical aspects that we considered for the design of the frequency tuning unit is the insulation of the electrical current, which may flow from the chevron thermal actuator to the electrostatically driven torsional resonator, resulting in a malfunction of the resonator. Preventing this charge leakage is another reason for employing the DSOI wafer with two isolated structural silicon layers, as shown in Figure 4. The thermal actuator is formed on the upper layer of the DSOI and the shuttle consists of both the upper and lower layers that are mechanically connected by the silicon oxide layer, but electrically isolated. When the thermal actuator is actuated, it makes contact only with the upper layer of the shuttle. However, the lower layer of the shuttle is also moved, since both layers of the shuttle are mechanically connected through the buried oxide, actuating the scissor mechanism that is defined on the lower layer. Thus, the connection of the tuning unit to the torsional shafts does not cause any charge leakage from the electrostatic actuator, since the only connection between the electrostatic resonator side and the thermal actuator side is through the buried oxide layer between the upper and lower layers of the shuttle.

## 3. Finite Element Analysis

**Figure 5:**
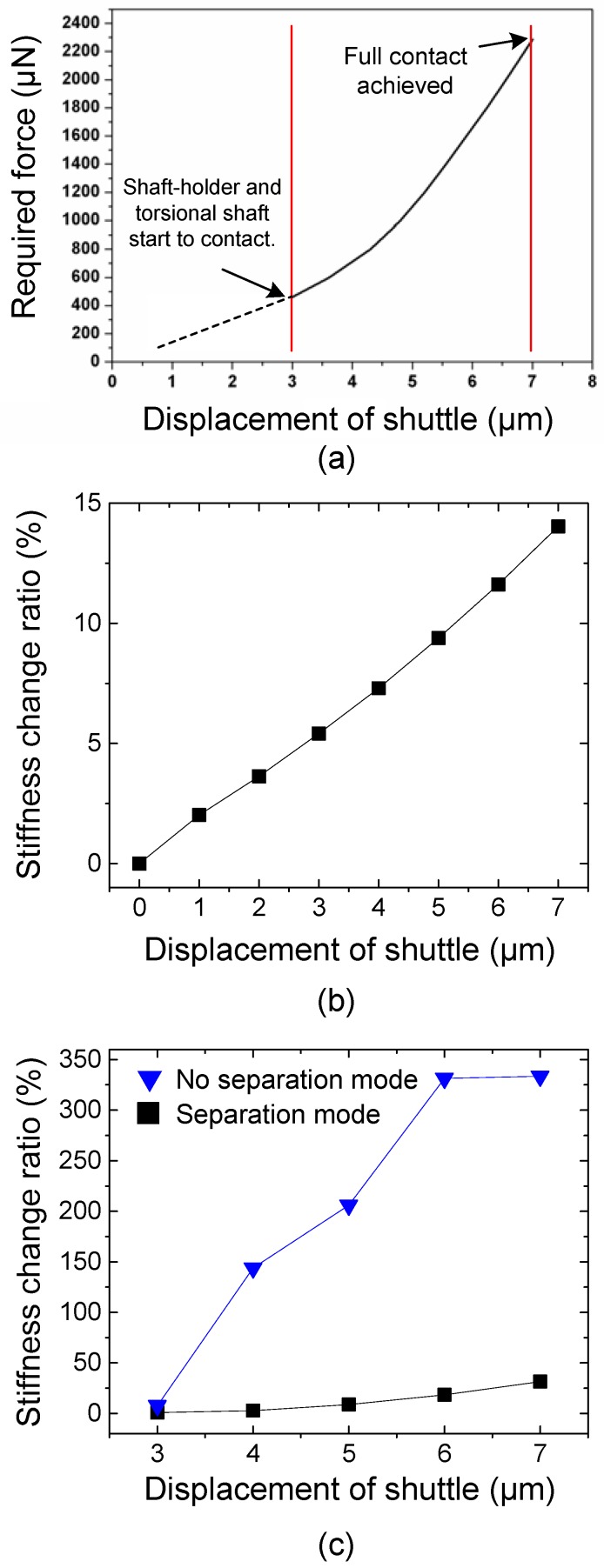
Finite element analysis (FEA) results: (**a**) Required force from the chevron thermal actuator to achieve full-contact between the shaft-holder and torsional shaft (shaft-holding type); (**b**) Estimated torsional stiffness change by shaft-widening type (**c**) Estimated torsional stiffness change by shaft-holding type. ‘No separation mode’ and ‘separation mode’ correspond to the restriction modes illustrated in Figure 6a,b, respectively.

For the design of the thermal chevron actuator, which may generate a limited amount of force, the stiffness of the scissor mechanism should be taken into consideration. Hence the finite element analysis (FEA) is conducted to calculate the required force from the thermal chevron actuator and to decide the proper dimensions of the scissor mechanism. According to the FEA results, the shaft-holder and torsional shaft start to contact with the thermal actuator stroke of 3 μm and make full contact at the stroke of 7 μm (Figure 5a) under the structural dimensions shown in Table 1. The required forces from the chevron thermal actuator for initial contact and full contact are 462 μN and 2286 μN, respectively (Figure 5a). Based on this finding, the chevron actuator is designed with the structural dimensions shown in Table 1.

In addition, the stiffness changes of the torsional shafts by the both tuning modes are quantified by FEA. First, modal analysis is performed for the shaft-widening type with the structural dimensions shown in Table 1. Based on the FEA result, we calculate torsional stiffness changes as a function of the displacement of the shuttle (i.e., stroke of the thermal actuator). As the shuttle is pulled by the thermal actuator, the torsional stiffness of the shaft gradually increases, as shown in Figure 5b. When the shuttle is moved by 7 μm, the torsional stiffness of the shaft increases by 14.0%, which corresponds to the tuning ratio of 3.4%.

**Figure 6:**
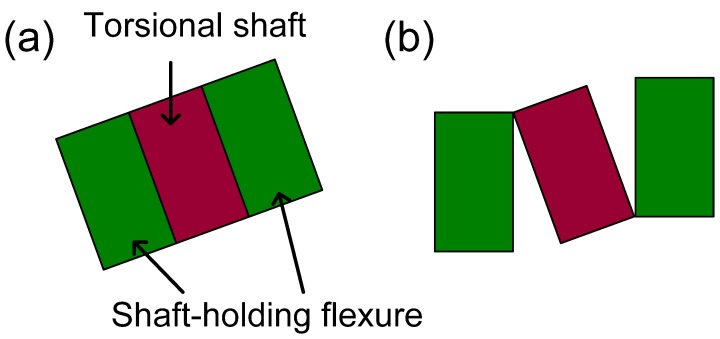
The cross-sectional view of the torsional shaft and shaft-holding flexure, showing two different restriction modes between the torsion bar and shaft-holding flexure. (**a**) No separation mode: the shaft-holding flexure is torsionally deformed together with the torsional shaft. The areal contact is maintained; (**b**) Separation mode: the areal contact is not maintained and the separation between the torsional shaft and shaft-holding flexure exist.

For analysis for the shaft-holding type, we supposed two different restriction modes that may possibly occur when the shaft-holding flexure makes contact with the torsion bar. The schematics in Figure 6 are the cross-sectional view of the torsional shaft and the shaft-holding flexure, showing the two restriction modes. In the first mode (Figure 6a), it is assumed that the shaft-holding flexure is torsionally deformed together with the torsion bar as if there is no slip or separation between the two structures (hereafter referred to as ‘no separation mode’). The FEA results for this boundary condition give a maximum stiffness change of 333.6% and a maximum resonant frequency increase of 63.3% compared to the original unrestricted structures (Figure 5b). The second mode, as depicted in Figure 6b, allows mechanical separation between the torsional shaft and the shaft-holding flexure, such that the mechanical restriction force is applied to the torsional shaft in the opposite direction of the shaft deformation, but the shaft-holding flexure is not torsionally deformed (hereafter referred to as ‘separation mode’). In this case, the maximum stiffness change is 31.6% and the maximum resonant frequency increase is 7.6%, showing the reduced restriction effect on the torsional shaft (Figure 5c). 

## 4. Fabrication

**Figure 7:**
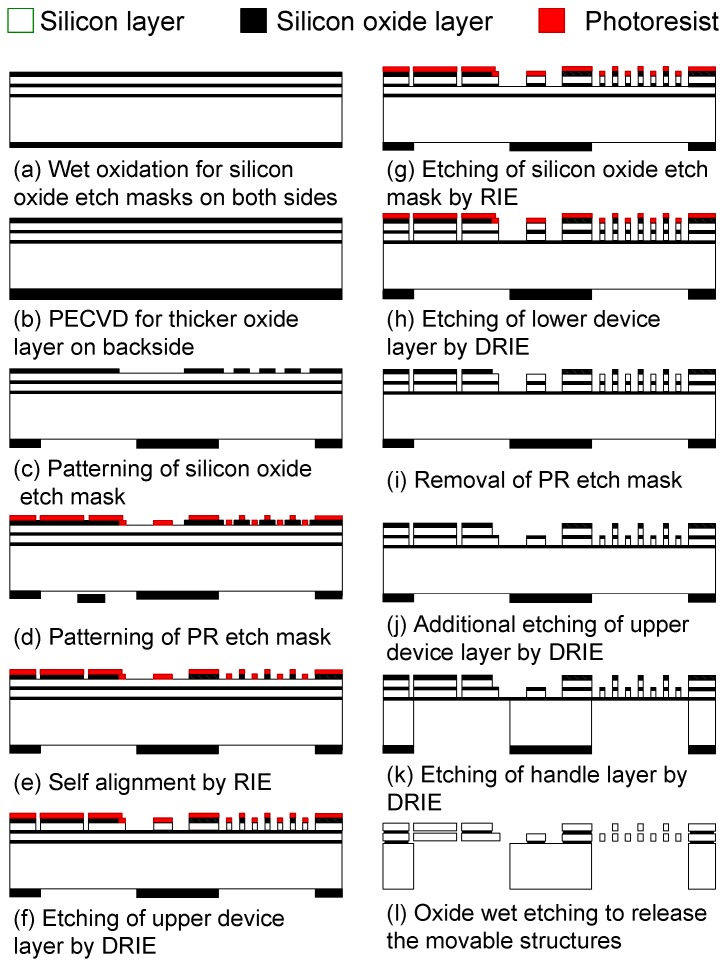
Fabrication process for the torsional resonator integrated with frequency tuning unit. The device is fabricated on a double silicon-on-insulator (DSOI) wafer. Reproduced with permission from [26].

Figure 7 illustrates the fabrication process for the torsional resonators and frequency tuning units [26]. Both of the structures are fabricated on DSOI wafer purchased from Ultrasil Corporation (Hayward, CA, USA). This wafer has two 20 μm-thick silicon device layers on the 425 μm thick base substrate, each of which is separated by buried oxide. The arsenic-doped silicon device layer has a resistivity less than 0.005 Ω·cm. To be used as etch masks, 1 μm-thick silicon oxide layers are grown on both the front- and backside of the wafer by wet oxidation process (Figure 7a). Additionally, a 2-μm-thick silicon oxide layer is deposited on the backside by plasma enhanced chemical vapor deposition (PECVD) to get a thicker backside etch mask (Figure 7b). Then the silicon oxide layers on both sides are lithographically patterned (Figure 7c). Next, the photoresist layer is patterned as shown in Figure 7d, which will be transferred to the lower device layer later. Between step (c,d), a rough alignment is acceptable, since the following oxide etch will form self-alignment between the silicon oxide and the photoresist layers (Figure 7e). After both of the device layers and the oxide layers are etched by deep reactive ion etching and reactive ion etching, respectively (Figure 7f–j), backside holes are defined (Figure 7k). Finally, to release the device, the remaining oxide etch masks and the buried oxide layers are wet-etched by exposing the entire chip to hydrofluoric acid (HF) solution (Figure 7l).

## 5. Result

**Figure 8:**
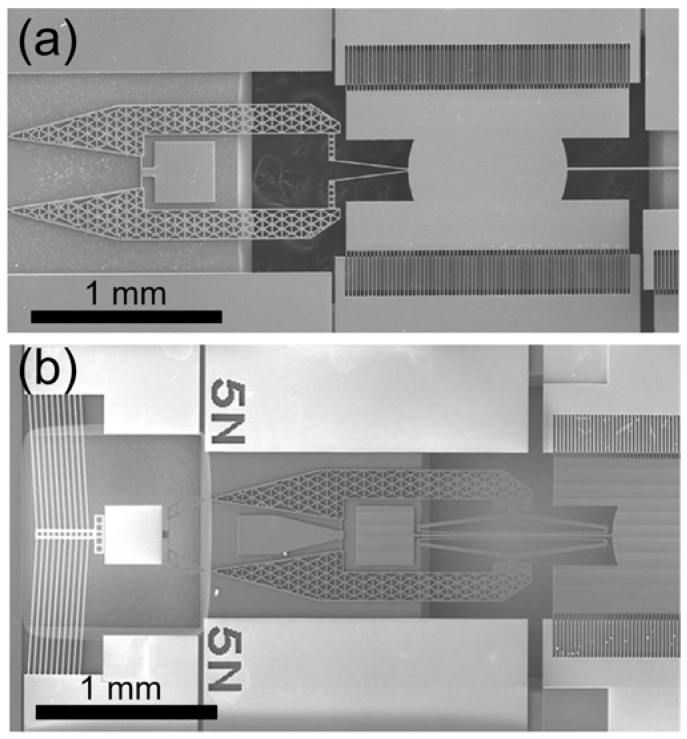
Scanning electron microscope (SEM) images of the fabricated devices: (**a**) Shaft-widening type and (**b**) shaft-holding type. Reproduced with permission from [26].

Figure 8a,b show scanning electron microscope (SEM) images of the fabricated devices for the shaft-widening type and the shaft-holding type [26]. The darker structures in the image are on the lower silicon layer, and the brighter structures are on the upper layer. The resonant frequency tuning of the electrostatic torsional resonator is experimentally verified while the driving voltages of 5 V_ac_ and 10 V_dc_ are applied on the resonator. To measure the rotational angle of the resonator, we focused a laser beam emitted from a laser diode on the micromirror of the resonator. The reflected laser beam scanned across a certain distance on a target screen. We measured the scanning distance of the laser beam (*R*), and calculated the rotational angle (*θ*) of the resonator as follows:

$$\theta =\tan^{-1}\left(\frac{R}{2D}\right)$$

where *D* is the distance between the target screen and the mirror. 

**Figure 9:**
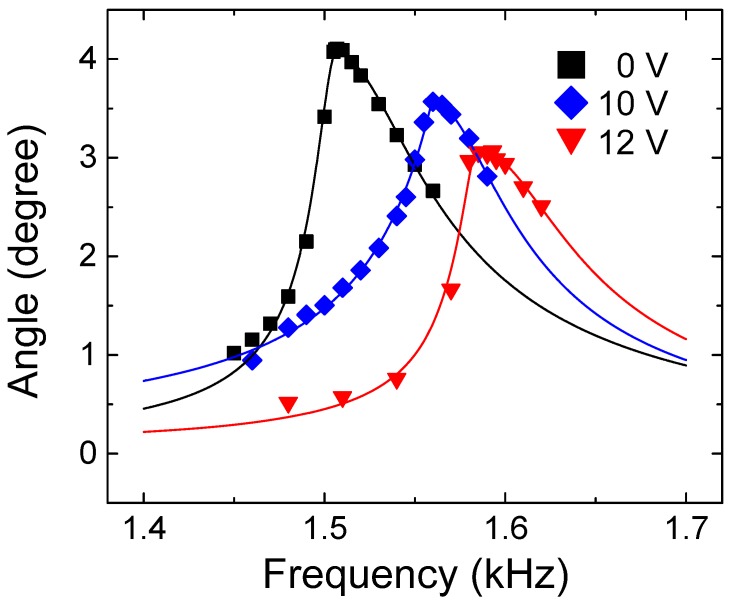
Frequency response change achieved by the shaft-widening type. Data points are fitted with the Lorentzian function.

Figure 9 shows how the frequency response of the resonator is changed from the untuned state to differently-tuned states by the shaft-widening type. The frequency response of our system does not perfectly comply with a form of Lorentzian curve; the stiffness-softening behavior is observed in all the frequency spectra, and is represented by a slight declination of the resonant peak toward the low frequency region. Due to this asymmetry, the left and right sides of the resonant frequency are fitted separately. We suspect that this nonlinearity mainly originated from the electrostatic actuation in our system [14]. However, obvious bifurcation or jump discontinuity due to the nonlinearity is not observed in the frequency spectra, thus we expect that the developed system will function well even in the presence of nonlinearity, as long as the excitation force no longer increases from the applied value. When the tuning DC voltages of 10 V and 12 V are applied to the shaft-widening type, the resonant frequency is shifted to 1.560 kHz and 1.593 kHz, respectively, from the untuned resonant frequency of 1.507 kHz (Table 2). The tuning ratios are 3.31% and 5.29% at tuning voltages of 10 V and 12 V, respectively. 

**Figure 10:**
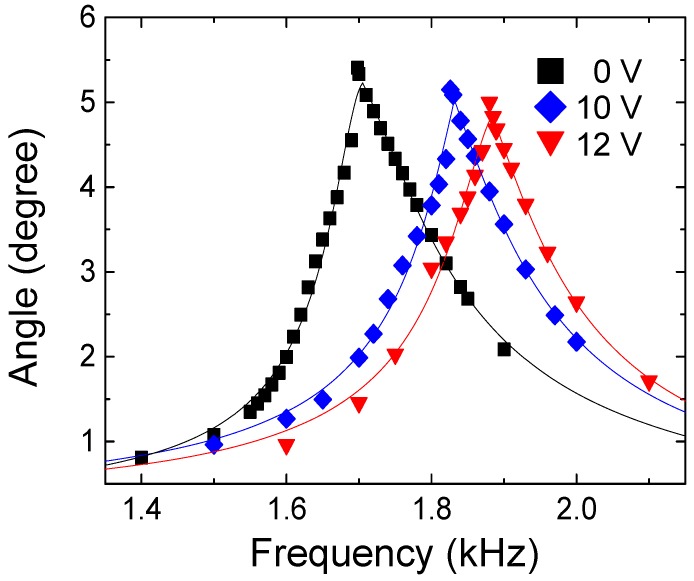
Frequency response change achieved by the shaft-holding type. Data points are fitted with the Lorentzian function. Reproduced with permission from [26].

In the shaft-holding type, when the tuning voltage of 7 V is applied to the thermal actuator, the shaft-holding flexures started to make contact with the torsional spring, and under 12 V, they formed full contact. Between 7 V and 12 V, the mechanical restriction process is completely reversible, and continuous frequency tuning up and down is achieved. The experimental measurement results for resonant frequency tuning are presented in Table 3, and changes in frequency responses are plotted in Figure 10 [26]. Under the tuning DC voltage of 8 V, 10 V and 12 V, the resonant frequency is shifted from the untuned resonant frequency of 1.698 kHz to 1.749 kHz, 1.826 kHz and 1.880 kHz, respectively, which correspond to the tuning ratio of 3.03%, 7.05% and 10.7%, respectively. The untuned resonant frequency of the torsional resonator (1.698 kHz) exhibits a deviation of 9.25% from the designed resonant frequency (1.871 kHz). The maximum tuning ratio of the shaft-holding type (10.7%) is sufficient to compensate for this discrepancy. However, it is noteworthy to mention that geometric error could be reduced under well-controlled fabrication conditions. The manufacturing processes for commercialized resonators probably have less fabrication errors than ours. In fact, as we mentioned earlier, it has been reported that the resonant frequencies of micromachined resonators generally exhibit a deviation between ±1%~±5% [7]. The Microfabrication Laboratory at UC-Berkeley have also reported a maximum deviation of 4.9% in resonant frequencies for batch-fabricated resonators [6]. Based on these criteria, the shaft-holding type, which exhibits lower tuning performance (maximum tuning ratio of 5.29%) than the shaft-holding type, also meets the needs of demand. It is expected that the tuning ratio would increase for the both types if the tuning actuator could be further operated, however, it could not be experimentally verified since the chevron thermal actuator burnt at a tuning voltage greater than 12 V.

**Figure 11:**
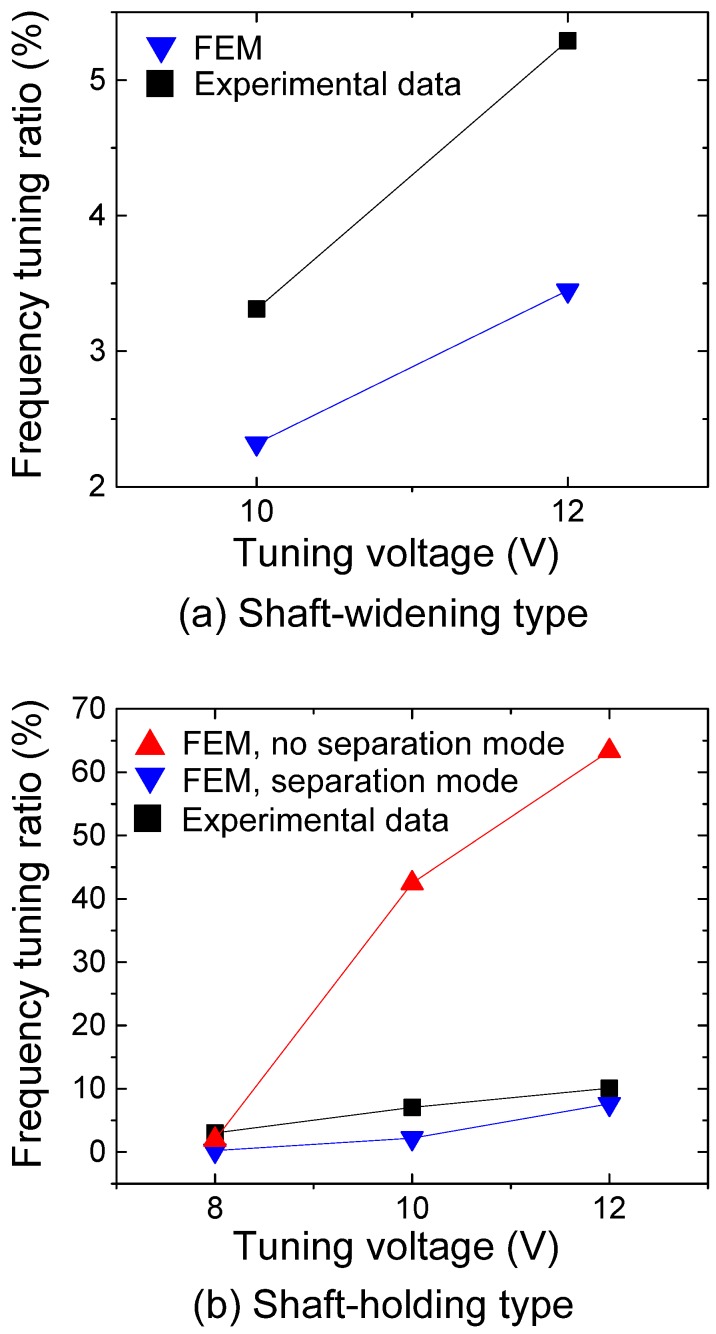
Measured and simulated results for resonant frequency change as a function of applied tuning voltage: (**a**) The shaft-widening type; (**b**) the shaft-holding type.

Figure 11 compares measured and simulated resonant frequency changes by the frequency tuning units. Both the measured and the simulated resonant frequencies increase with the application of tuning voltage, but there is some discrepancy in values between them. For example, the simulated values for the shaft-widening type differ from the experimental results up to 34.8% (Figure 11a). Our study does not further verify the factors causing this discrepancy. However, it is worth exploring possible sources. Dimensional error from the microfabrication process could be one of the sources of this mismatch. Imperfect lithography and the ion-reactive process might change the performance of the tuning unit, if the resonant frequency is shifted from the desired value. It is also worth noting that we used simplified boundary conditions for FEA. For example, when we derive the resonant frequency by modal analysis, we do not consider some conditions that may cause nonlinearity, including relatively large deflection, sliding between contact surfaces, varying contact area, and the electrostatic spring effect.

In the previous section, we assumed two restriction modes in the shaft-holding type: no separation and separation modes. At a tuning voltage of 12 V, the estimated frequency tuning ratios for the no separation and separation mode are 63.3% and 7.6%, respectively (Figure 11b). It is not obvious which mode is really happening, and a co-existence of both modes is also possible. However, the frequency tuning ratio of the no separation mode is closer to that of the experimental results (10.7% at 12 V).

Since our tuning mechanism is based on mechanical stress and contact, we have extracted Q-factors for each frequency spectrum. Instead of a half-power bandwidth formula, which is generally used to calculate Q-factors for linear oscillating system, we used an alternative formula that is modified for a non-linear system, especially for a micro-scanning mirror [29]. The Q-factors of the shaft-widening type are calculated as 28, 27, and 29 at applied tuning voltages of 0 V, 10 V, and 12 V, respectively. Considering that the Q-factor remains almost constant as the tuning is applied, it seems that the level of energy dissipation does not change significantly with the increment of the stress inside the spring. The Q-factors of the shaft-holding type are 17, 16, and 16 at applied tuning voltages of 0 V, 10 V, and 12 V, respectively, showing a slight decrease even under mechanical restriction. One of the possible scenarios explaining this is that the resonator is under the dominant influence of the air damping, since a relatively large mass (i.e., the micromirror) is oscillating in the ambient environment. Thus, the contribution of mechanical stress or contact to the overall energy dissipation may not be notable.

## 6. Conclusions

In this work, the resonant frequency tuning of a torsional resonator has been demonstrated by two concepts: shaft-widening and shaft-holding. Based on the FEA results, we verified the validity of our tuning methods, and derived the estimated change of torsional stiffness and tuning range. The frequency tuning units were integrated into the resonator, and there is no necessity for additional post fabrication processes for frequency-tuning. We experimentally verified the performance of both designs. The results indicate that the shaft-holding type has a wider tuning range compared to the shaft-widening type. Nevertheless, the tuning ratios of both are higher compared to those of the previously method [14], and are also sufficient to compensate for the dimensional errors of the conventional microfabrication process [6,7].

## Figures and Tables

**Table 1:** Design parameters for the torsional resonator and tuning unit.

**Torsional Resonator**	Mirror diameter	800 μm
	Length/width of straight shaft	1050 μm /10 μm
	Length/width of tilted shaft	600 μm /10 μm
	Length/width of Comb	200 μm /5 μm
	The number of moving/fixed Comb	72/73
	Thickness	20 μm
**Scissor Mechanism**	Total length/width	2240 μm /680 μm
	Length/width of shuttle	300 μm /300 μm
	Length/width of hinges	60 μm /5 μm
**Shaft-Holder**	Total length	1000 μm
	Width of shaft-holding flexure	5 μm
**Chevron Thermal Actuator**	Length/width of chevron beam	700 μm /10 μm
	Number of chevron beam	20
	Angle of chevron beam	1.06°

**Table 2:** Resonant frequency shifts achieved by the shaft-widening type.

Tuning Voltage (V)	Resonant Frequency (kHz)	Tuning Ratio (%)
0 V	1.507	-
10 V	1.560	3.31
12 V	1.593	5.29

**Table 3:** Resonant frequency shifts achieved by the shaft-holding type. Reproduced with permission from [26].

Tuning Voltage (V)	Resonant Frequency (kHz)	Tuning Ratio (%)
0 V	1.698	-
8 V	1.749	3.03
10 V	1.826	7.05
12 V	1.880	10.7

## References

[1] Zhang W.M., Hu K.M., Peng Z.K., Meng G. (2015). Tunable micro- and nanomechanical resonators. Sensors.

[2] Toshiyoshi H., Fujita H. (1996). Electrostatic micro torsion mirrors for an optical switch matrix. J. Microelectromech. Syst..

[3] Hwang K.S., Lee S.M., Kim S.K., Lee J.H., Kim T.S. (2009). Micro- and nanocantilever devices and systems for biomolecule detection. Annu. Rev. Anal. Chem..

[4] Madou M.J. (2012). Fundamentals of Microfabrication and Nanotechnology.

[5] Tang W.C., Nguyen T.-C.H., Judy M.W., Howe R.T. (1990). Electrostatic-comb drive of lateral polysilicon resonators. Sens. Actuators A Phys..

[6] Chiao M., Lin L. (2004). Post-packaging frequency tuning of microresonators by pulsed laser deposition. J. Micromech. Microeng..

[7] Hsu W.-T., Brown A.R. Frequency trimming for mems resonator oscillators. Proceedings of the IEEE International Conference on Frequency Control Symposium.

[8] Yong Y.-K., Vig J.R. (1989). Resonator surface contamination—A cause of frequency fluctuations?. IEEE Trans. Ultrason. Ferroelectr. Freq. Control.

[9] Koskenvuori M., Mattila T., Häärä A., Kiihamäki J., Tittonen I., Oja A., Seppä H. (2004). Long-term stability of single-crystal silicon microresonators. Sens. Actuators A Phys..

[10] Enderling S., Hedley J., Jiang L.D., Cheung R., Zorman C., Mehregany M., Walton A.J. (2006). Characterization of frequency tuning using focused ion beam platinum deposition. J. Micromech. Microeng..

[11] Joachim D., Lin L. (2003). Characterization of selective polysilicon deposition for mems resonator tuning. J. Microelectromech. Syst..

[12] Syms R.R.A., Moore D.F. (1999). Focused ion beam tuning of in-plane vibrating micromechanical resonators. Electron. Lett..

[13] Witkamp B., Poot M., Pathangi H., Hüttel A., Van der Zant H. (2008). Self-detecting gate-tunable nanotube paddle resonators. Appl. Phys. Lett..

[14] Eun Y., Kim J., Lin L. (2014). Resonant-frequency tuning of angular vertical comb-driven microscanner. Micro Nano Syst. Lett..

[15] Lee W.-S., Kwon K.-C., Kim B.-K., Cho J.-H., Youn S.-K. (2004). Frequency-shifting analysis of electrostatically tunable micro-mechanical actuator. CMES Comp. Model. Eng. Sci..

[16] Gallacher B.J., Hedley J., Burdess J.S., Harris A.J., Rickard A., King D.O. (2005). Electrostatic correction of structural imperfections present in a microring gyroscope. J. Microelectromech. Syst..

[17] Lee K.B., Cho Y.-H. (1998). A triangular electrostatic comb array for micromechanical resonant frequency tuning. Sens. Actuators A Phys..

[18] Lee K.B., Lin L., Cho Y.-H. (2008). A closed-form approach for frequency tunable comb resonators with curved finger contour. Sens. Actuators A Phys..

[19] Jensen B.D., Mutlu S., Miller S., Kurabayashi K., Allen J.J. (2003). Shaped comb fingers for tailored electromechanical restoring force. J. Microelectromech. Syst..

[20] Morgan B., Ghodssi R. (2008). Vertically-shaped tunable mems resonators. J. Microelectromech. Syst..

[21] Scheibner D., Mehner J., Reuter D., Kotarsky U., Gessner T., Dötzel W. (2004). Characterization and self-test of electrostatically tunable resonators for frequency selective vibration measurements. Sens. Actuators A Phys..

[22] Remtema T., Lin L. (2001). Active frequency tuning for micro resonators by localized thermal stressing effects. Sens. Actuators A Phys..

[23] Syms R.R. (1998). Electrothermal frequency tuning of folded and coupled vibrating micromechanical resonators. J. Microelectromech. Syst..

[24] Elata D., Leus V., Hirshberg A., Salomon O., Naftali M. A novel tilting micromirror with a triangular waveform resonance response and an adjustable resonance frequency for raster scanning applications. Proceedings of the TRANSDUCERS Solid-State Sensors, Actuators and Microsystems Conference.

[25] Shmilovich T., Krylov S. Linear tuning of the resonant frequency in tilting oscillators by an axially loaded suspension flexure. Proceedings of the IEEE 21st International Conference on Micro Electro Mechanical Systems.

[26] Lee J.-I., Park S., Eun Y., Jeong B., Kim J. Resonant frequency tuning of torsional microscanner by mechanical restriction using mems actuator. Proceedings of the IEEE 22nd International Conference on Micro Electro Mechanical Systems.

[27] Ya’akobovitz A., Krylov S., Shacham-Diamand Y. (2008). Large angle SOI tilting actuator with integrated motion transformer and amplifier. Sens. Actuators A Phys..

[28] Lott C.D., McLain T.W., Harb J.N., Howell L.L. (2002). Modeling the thermal behavior of a surface-micromachined linear-displacement thermomechanical microactuator. Sens. Actuators A Phys..

[29] Davis W.O. (2011). Measuring quality factor from a nonlinear frequency response with jump discontinuities. J. Microelectromech. Syst..

